# Body Composition and Its Interaction with Bone Mineral Density and Biochemical and Nutritional Parameters in Chilean Adults with Overweight/Obesity and Normal Weight

**DOI:** 10.3390/nu16111559

**Published:** 2024-05-21

**Authors:** Francisca Villagrán-Silva, Fernando Lanas, Nolberto Huard, Luis A. Salazar

**Affiliations:** 1 Doctoral Program in Morphological Sciences, Faculty of Medicine, Universidad de La Frontera, Temuco 4811230, Chile; f.villagran04@ufromail.cl; 2 Center of Molecular Biology and Pharmacogenetics, Department of Basic Sciences, Faculty of Medicine, Universidad de La Frontera, Temuco 4811230, Chile; lanastomas@gmail.com (F.L.); n.huard@ufrontera.cl (N.H.); 3 Department of Internal Medicine, Faculty of Medicine, Universidad de La Frontera, Temuco 4811230, Chile

**Keywords:** anthropometric, body composition, overweight/obesity, bone mineral density, lipid profile, glucose, physical activity, diet

## Abstract

This study aimed to compare and relate the body composition (obtained through anthropometry with the pentacompartmental model and the tricompartmental model by DXA) with bone mineral density and biochemical and nutritional parameters in Chilean adults with overweight/obesity and normal weight from La Araucanía region, Chile. A case-control study was conducted with 116 adults and volunteers from the PURE cohort, collecting sociodemographic data, BMI assessment, waist-to-hip ratio (WHR), and body composition using the pentacompartmental model (5CM) and tricompartmental model (3CM) by DXA, as well as bone mineral density (BMD). Blood biochemical parameters (fasting glucose and lipid profile), physical activity (PA) measured by GPAQ, and average dietary habits (R24h) were measured. In the overweight/obesity group, the 5CM and 3CM adipose mass were indirectly and moderately correlated with PA (*p* < 0.05), except in the male 5CM group. In the overweight/obesity group, muscle and fat-free mass (FFM) of the 5CM and 3CM correlated directly and moderately with blood fasting glucose (BFG) and BMD (*p* < 0.05), except in females, where FFM was not related to BMD but was related to residual mass (*p* < 0.01). Independent of gender and BMI, bone mineral content was positively and highly correlated with BMD (*p* < 0.0000). In the male overweight/obesity group, bone, skin, and residual mass were correlated with BFG (*p* < 0.05). In conclusion, for the assessment of non-athletic adult populations, more routine use of the 5CM in clinical practice is recommended.

## 1. Introduction

One of the fastest-growing metabolic diseases, considered a new epidemic, is overweight and obesity, which is generated by an energetic imbalance between the consumed and expended calories with an increase in body fat [1,2,3,4]. Consequently, there is a shorter life expectancy due to a higher risk of acquiring numerous diseases, such as insulin resistance, type 2 diabetes mellitus, cardiovascular diseases, osteoarticular pathologies, sleep apnea, and other respiratory problems, besides certain types of cancer (endometrium, breast, ovary, prostate, liver, gallbladder, kidney, and colon) [1,3,4,5,6].

Obesity is a public health problem; in 2016, globally, more than 1.9 billion adults aged 18 or older (39.0%) were overweight (39.0% of men and 40.0% of women), of which over 650 million were obese (around 13.0%; 11.0% men and 15.0% women) [2]. These figures are alarming and rising in low- and middle-income countries, particularly in urban environments [2]. Chile is positioned in first place, having the highest rate of overweight and obesity at 74.2% of its population, surpassing Mexico (72.5%) and the United States (71.0%) [7]. Specifically, the Araucanía region is one of those with the highest rates of adult obesity in the country, with 34.5% [8].

Body composition assessment is needed to estimate the risk of excess adiposity and prevent the development of comorbidities associated with overweight, obesity, and the risk of musculoskeletal injuries in adults [9,10]. There are three types of methods to estimate body composition: direct method, through cadaver dissection, and indirect method, with sophisticated methods such as nuclear magnetic resonance, computed tomography, air displacement plethysmography, isotope dilution method, and bioelectrical impedance analysis, which are not cost-effective and present difficulties in analyzing nutritional status reports. Some, as a result of high radiation levels, end up affecting their health with their repeated use [11]. An exception is dual X-ray absorptiometry (DXA), which is less expensive, uses low-intensity radiation, and is commonly used in a clinical context to quantify the total, lumbar, and femoral head bone mineral density (BMD) to diagnose osteopenia and in more severe cases, such as osteoporosis [11,12,13]. It also provides an exhaustive validated report to assess body composition in images, including body masses (fat, lean, and bone mineral) of the complete body and by segments, which is considered a tricompartmental model (3CM) [11,13,14].

Doubly indirect methods are obtained from anthropometric measurements. One of the most commonly used tools worldwide to conduct comparative population-based epidemiological analysis of nutritional status is the body mass index (BMI) [15,16]. However, BMI does not distinguish between muscle and fat mass, leading to an overestimation of nutritional status, and does not determine the distribution of adiposity [16,17]. On the other hand, another method for estimating body composition in the clinical and epidemiological settings with anthropometric measurements is the bicompartmental model (fat mass and free-fat mass) [15], which is low-cost and corresponds to reliable estimates for measuring nutritional status and the degree of obesity precisely [16,18,19]. In this context, although the bicompartmental model is more practical in clinical use because it requires fewer body measurements, more than 150 linear regression model formulas have been generated; however, these formulas can only be extrapolated if the sample meets the same fitting conditions [20]. The pentacompartmental model (5CM) offers a solution to this problem. It consists of five components: adipose mass, muscle mass, bone mass, residual mass (viscera and organs), and skin mass.

The 5CM represents an enhanced version of the tetracompatmental model, having undergone validation across various gender and age groups in both good and poor physical conditions [21]. This method figures out the body proportionality of each body segment, which is called the Phantom [21]. It has been shown to be accurate with tissue masses obtained through dissection in the study of Brussels cadavers [22] and in 12,282 men and 6593 women aged 6 to 77 years [23]. It is commonly used in general and elite sports [24,25] because it provides the complete anthropometric profile and the differentiation of body masses, which is of great importance for comparing athletic biotypes in different populations.

Although the literature presents studies on the interaction between body composition measured using DXA, bone mineral density, nutritional status, and physiological parameters in non-athletic adult populations [26,27,28,29,30], the literature does not present studies in the clinical setting of 5CM. Therefore, it is necessary to show a new alternative for measuring body composition with reliable estimates, a more complete report, lower cost, and greater accessibility for use at a clinical and epidemiological level. Thus, in this study, we aimed to compare and correlate body composition (obtained with the 5CM and 3CM) and its interaction with bone mineral density and biochemical and nutritional parameters (dietary habits and physical activity) in Chilean adults with overweight/obesity and normal weight.

## 2. Materials and Methods

### 2.1. Study Design and Subjects

This quantitative, analytical, non-experimental, observational, case-control study was carried out on Chileans from the region of La Araucanía, specifically from urban and rural areas of the commune of Temuco. We randomly selected the sample from 3500 adults participating in the annual follow-up study of the Prospective Cohort of Urban Rural Epidemiology (PURE) at the Center for Cardiovascular Studies of the Universidad de La Frontera and extended an open invitation to volunteers via social networks. Participants had to meet the following inclusion criteria: adults between 45 and 59 years of age of both genders. They were divided into two groups: a control group with normal weight (BMI between 18.5 kg/m^2^ and 24.9 kg/m^2^) and a case group with overweight and obesity (BMI between 25.0 kg/m^2^ and 34.9 kg/m^2^). We excluded participants based on the following criteria: 1. BMI ≥ 35.0 kg/m^2^ (type II and III obesity) because the anthropometric instruments are not calibrated to measure subjects with skinfold calipers greater than 60 mm and weight greater than 120 kg, which exceeded the acceptable weight limit of the DXA machine; 2. BMI < 18.5 kg/m^2^ (underweight); 3. weight loss surgery (gastric sleeve, gastric bypass, and adjustable gastric band); 4. diseases such as cancer and atherosclerosis; and 5. physical disabilities.

The sample consisted of 116 subjects out of a total of 122 participants. All procedures except for bone densitometry (DXA) were performed at the Cardiovascular and Nutritional Epidemiology Research Center (EPICYN) of the Universidad de La Frontera from December 2021 to December 2022. Due to the global and national health situation caused by the SARS-CoV-2 virus infection, all preventive and sanitation measures were taken for each participant.

### 2.2. Measurements and Instruments

#### 2.2.1. General Survey

We explained the protocol and the informed consent to each participant. After the acceptance into the study, a survey was conducted to obtain personal data and biosociodemographic characteristics such as age, sex, geographic area, employment status, marital status, number and type of chronic noncommunicable disease (NCD), and medication consumption.

#### 2.2.2. Anthropometric Measurements

The methodology for the anthropometric evaluation was performed during the first hours of the morning. In addition, the following recommendations were provided for each participant in the informed consent: fasting for no more than 12 h after urinating and wearing light clothing (men with Lycra shorts and women with swimsuits). The 23 anthropometric measurements were performed in duplicate and recorded on an evaluation form based on the international protocol for anthropometric assessment [31] performed using an ISAK level 3 accredited anthropometrist (F.V.S), with a duration of 30 min per participant.

Body weight was measured using a SECA^®^ model 803 digital scale (Seca GmbH & Co. KG, Hamburg, Germany), with an accuracy of 0.1 kg and a capacity of 150 kg. Height was measured using a SECA^®^ model 213 detachable and portable stadiometers with an accuracy of 0.1 cm and a measuring range of up to 220 cm. Sitting height was measured using a standardized anthropometric box (30 cm in width, 50 cm in length, and 40 cm in height) with a measuring range of up to 120 kg and the SECA model 213 stadiometer (Seca GmbH & Co. KG, Hamburg, Germany). Eight circumference measurements (head, relaxed arm, forearm maximum, mesoesternal thoracic, waist (minimum), hip (maximum), thigh (medial), and calf (maximum)) were taken using a Lufkin WP-606 (Lufkin, TX, USA) steel tape, with a measuring range of up to 200 cm and an accuracy of 0.1 cm. The four large bone diameters (biacromial, transverse thoracic, anteroposterior thoracic, and bi-iliocrestal) were measured using a Rosscraft^®^ Campbell 20 sliding anthropometer (Rosscraft, Surrey, BC, Canada), with a measuring range of 54 cm and an accuracy of 0.1 cm. The two small bone diameters (humeral and femoral) were measured using a Rosscraft^®^ Campbell 10 sliding anthropometer (Rosscraft, Surrey, BC, Canada), with a measuring range of 15 cm and an accuracy of 0.1 cm. The six skinfolds (triceps, subscapular, supraspinatus, abdominal, mid-thigh, and leg (maximum)) were measured with a Slim Guide caliper (Cescorf, Porto Alegre, Brazil), with a maximum measurement range of 85 mm and a sensitivity of 1 mm.

#### 2.2.3. Anthropometric Indices and Body Composition

The following anthropometric indices formulas were used to describe the adiposity of the sample:

- Body mass index (BMI) = Weight (kg)/Height^2^ (m)
- Waist–hip ratio (WHR) = WC (cm)/HC (cm) [32]

Twenty-two anthropometric measurements (excluding hip circumference) were used to calculate body composition using the pentacompartmental model (5CM), which assesses fat, bone, muscle, skin, and residual mass [23], using ISAK Metry software (https://isakmetry.com/). Fat-free mass was calculated as muscle, fat, bone, and skin mass. All masses were expressed in kg and percent. The 5CM has been validated for predicting body mass in 1669 subjects representing 11 groups of men and women, which included older and younger adults, individuals in good or poor physical condition, and individuals with a wide range of habitual physical activity. Additionally, its accuracy has been confirmed through comparison with tissue masses obtained from dissection conducted in the study of Brussels cadavers in a sample of 12 men and 13 women. Both criteria gave correlations of 0.93 between the mass predicted from the sum of the five masses and the mass obtained, except in light rowers, with a reduced variance with respect to the mean (in any case, it should be noted that the sample of rowers had the lowest standard errors of estimated weight). This method had mean errors in the prediction of the total mass of 2.2% and 3.4% for women and men, respectively, with values below 5% being established as the reliability limit [18].

The clinical tricompartmental (3CM) body composition clinical examination of fat mass, lean mass, and bone mineral content (BMC) or bone mineral mass and bone mineral density (BMD) was conducted using equipment the Lunar Prodigy DXA System (v. 13.20, GE Healthcare, Chicago, IL, USA). All masses were considered in kg and percentage. In addition, fat-free mass was considered the sum of lean mass and BMC. BMD is the calculation of the ratio of BMC per unit area (g/cm^2^), comparing the values obtained to the reference database, T-score, and Z-score. The T-score is used to diagnose osteoporosis in postmenopausal women and men aged ≥50 years. The T-score is defined as the number of standard deviations between the patient’s BMD and the mean of a young adult reference population of the same gender. A T-score > −1.0 is considered normal, a T-score between −1 and −2.5 indicates osteopenia and a T-score < −2.5 indicates osteoporosis. The Z-score is used for premenopausal women, men under the age of 50, and children and adolescents (up to the age of 20). The Z-score is defined as the number of standard deviations (SDs) between the patient’s BMD value and the mean of a reference population of the same race, gender, and age. If the Z-score is less than −2 SD, the diagnosis is low bone density due to age. The accuracy of DXA is high, with a margin of error of 2–6% for body composition [12,13].

#### 2.2.4. Biochemical Analysis

Lipid parameters (total cholesterol, triglycerides, HDL-cholesterol, and LDL-cholesterol) and blood fasting glucose (BFG) were obtained from blood samples, with a fasting period of no more than 12 h. The procedure was carried out by the nursing technician of the PURE cohort in a specific box for blood samples at EPICYN. The samples were placed in an 8 mL tube with separator gel (yellow cap), labeled with the participant’s code and date, for posterior centrifugation at 3000 rpm for 15 min, serum extraction, and refrigerated storage at +4 °C. The collected samples were transferred to the Molecular Biology and Pharmacogenetics Center of the Universidad de La Frontera in a refrigerator with cooling gel and stored at −80 °C to maintain sample stability.

The analysis was carried out with the enzymatic colorimetry technique of commercial kits in a semi-automatic photometer (Humalyzer 3000, Wiesbaden, Germany), which was previously calibrated and subjected to quality control, with all the biosafety measures during processing. Samples were gradually thawed (from −80 °C to −20 °C for 12 h, at +4 °C for 4 h, and at room temperature for 1 h) before use. Furthermore, the reagents were stored at 4 °C to maintain stability and avoid contamination.

The following ranges were considered: normal BFG between 75–110 mg/dL, with values >110 mg/dL considered at-risk; normal total cholesterol between 125 and 200 mg/dL, with the upper limit of the normal range falling between 200 and 239 mg/dL, and those >240 mg/dL considered at-risk; normal LDL-cholesterol ≤130 mg/dL, with values >130 mg/dL considered at-risk; normal HDL-cholesterol >50 mg/dL for women, with values ≤50 mg/dL considered at-risk, and normal >40 mg/dL for men, with ≤40 mg/dL considered at-risk; and normal triglycerides <150 mg/dL, with ≥150 mg/dL considered at-risk.

#### 2.2.5. PA and R24h Survey

We measured the subject’s physical activity (PA) habits using the World Health Organization’s Global Physical Activity Questionnaire (GPAQ) and its corresponding analysis guide [33]. To determine the intensity of the physical activity of the subjects, whether sedentary, moderate, or vigorous, we used the calculation of MET min/week, which is the unit of measurement of the metabolic rate (the ratio between a person’s metabolism during the performance of work and his or her basal metabolism).

A 24 h food reminder questionnaire (R24h) was conducted via video call with a trained nutritionist, considering three reminders (two on weekdays, every other day, and one on weekends) to obtain the average number of calories consumed in one week. The R24h multi-step method (R24MP) of the United States Department of Agriculture (USDA) method was applied: 1—fast listing of foods and beverages consumed during the day; 2—forgotten foods; 3—time and time of meal (e.g., breakfast, lunch, dinner, snack, etc.); 4—details of foods in the pre-meals and portion sizes; and 5—final review as a quick review of the food listing [34]. To assist the participants in the definition of portions, the interviewer used the photographic atlas of the ENCA 2010 [35].

### 2.3. Statistical Analysis

Statistical analyses were performed using STATA^®^ version 18 software. Distributions were determined using the Shapiro–Wilk test. In this study, using descriptive and inferential statistics, the obtained results were displayed in percentage and frequency for nominal and ordinal variables; for continuous variables with normal distribution, they were presented in mean and standard deviation (SD); and for those that were not normally distributed, they were presented in median and interquartile range (IQR). To compare biosociodemographic parameters, PA levels, WHR, and BMD categories by gender and BMI, the Chi^2^ test was applied when the frequency was greater than 5 per group; otherwise, Fisher’s exact test was applied. Independent sample *t*-Student tests by gender and BMI were performed to determine age differences, R24h, biochemical parameters, anthropometric profile, WHR, body composition measured by 5CM and 3CM, and BMD. The Mann–Whitney U test was used for PA, with values described as median and IQR. The correlation between body composition by 5CM and 3CM, biochemical and nutritional parameters, and BMD by gender and BMI was assessed using Pearson’s correlation coefficient (r) and PA with Spearman’s rank correlation (rho). All statistical tests were analyzed with a significance level of α = 0.05.

### 2.4. Ethical Statement

Regarding ethical considerations, the present study was approved by the Scientific Ethical Committee of the Universidad de La Frontera, Protocol Number 095_21, with the protocol and informed consent forms being reviewed.

The participants reviewed the written protocol and the informed consent, resolving any doubts with the responsible investigator. This consent form included information on the purpose of the study, the benefits and risks of the protocols to be applied (anthropometric measurements, blood sampling, and clinical examination of bone densitometry), and the voluntary nature of participation. They were informed that they could refuse or withdraw from the research at any time.

We assigned the participants a code to protect the privacy and confidentiality of their personal data. Only the responsible researcher can identify the subject thanks to this procedure that unlinks the participant’s personal data.

## 3. Results

### 3.1. General Information about Participants

Table 1 shows the biosociodemographic characteristics and nutritional and biochemical parameters of normal-weight adults and those with overweight or obesity classified by gender. Regarding sociodemographic characteristics such as location, employment situation, and marital status, there were no significant differences between normal weight and overweight or obesity based on gender. The men with overweight or obesity showed significantly higher levels of BFG, LDL and HDL cholesterol, and triglycerides compared with the normal weight. Women with overweight or obesity showed significantly higher levels of BFG and triglycerides compared with the group of normal-weight women.

### 3.2. Anthropometric Profile, Anthropometric Index, and Body Composition

Table 2 shows the anthropometric profile of normal weight and overweight/obese men and women, where we can see that men with overweight/obesity have significant differences in weight measurements, four diameters (biacromial, transverse chest, anteroposterior chest, and bi-iliocrestidial), the totality of circumferences, and four skinfolds (triceps, subscapular, supraspinal, and abdominal) in comparison with the group of normal-weight men. It is important to highlight that those men with malnutrition have an excess of 15 kg of body weight, are almost 10 cm more in waist and hip circumference, and are almost 10 cm larger than men with a healthy nutritional status.

Table 3 compares the anthropometric indices and body composition measured using the pentacompartmental model (5CM) and the tricompartmental model (3CM) and bone mineral density between normal-weight adults and those with overweight or obesity both in men and women. It is evident that there is the same significant difference in the mean WHR of 0.06 between men and women. Furthermore, 63.6% (*n* = 21) and 90.9% (*n* = 30) of men and women, respectively, belonging to the group overweight or obesity were in the category WHR index of excessive fat and obesity. The body composition measured using the 5CM and 3CM showed significant differences in the mean kg of each body mass between the groups with overweight/obesity and normal weight in both sexes, except for the entire body bone mineral content (BMD) in men. The body masses (kg) were higher in the group with overweight/obesity. The group with a higher mean BMD was the group with overweight/obesity in both genders.

### 3.3. Relationship between Body Composition by 5CM and 3CM and Biochemical, Nutritional Parameters, and BMD

In Table 4 and Table 5, significant correlations of body composition by 5CM and 3CM, respectively, among biochemical and nutritional parameters and BMD are shown in adult men who are overweight/obese. Evidence reveals a significant direct and moderate correlation between BFG and muscle mass, bone mass, skin mass, residual mass, and FFM and BMD with muscle mass and FFM, as measured by 5CM (Table 4 and Figure S1a–g). A similar phenomenon occurred with 3CM, showing a significant, direct, and moderate correlation between BFG and lean mass, FFM, between BMD and lean mass and FFM; and between BMD and BMC. On the other hand, Table 5 and Figure S2a–f show that only fat mass measured using 3CM significantly, indirectly, and moderately correlates with PA.

In the group of women who are overweight/obese, Table 4 reveals a direct and moderately significant correlation between BFG and muscle mass, as well as FFM (see Figure S3a,b); between blood triglyceride levels and bone mass (Figure S3c); between BMD and muscle mass (Figure S3d); and indirect and moderate PA and adipose mass (Figure S3e), as well as residual mass (Figure S3f), as measured by 5CM. In the 3CM shown in Table 5, a direct and moderately significant correlation is evidenced between BFG and lean mass (Figure S4a), as well as FFM (Figure S4b), and between BMD and lean mass (Figure S4c), and FFM (Figure S4e), and a high correlation between BMD and BMC is shown (Figure S4d). In addition, fat mass was indirectly and moderately correlated with PA (Figure S4f).

## 4. Discussion

In the present study, we evidenced that, regardless of gender, there are significant differences in body composition measured by the anthropometric method of 5CM and 3CM using DXA between the overweight/obesity group and the normal weight group, except for total body BMD in men. A body fat percentage greater than 25% (considered obesity) in normal weight and overweight/obese men, measured using both methods, confirms the overestimation of BMI. The WHR and BMD were higher in the overweight/obesity group. Regarding blood biochemical parameters, the overweight/obesity group of men had average above normal ranges in BFG, LDL cholesterol, and HDL cholesterol, with triglycerides being notably elevated, as indicated by the standard deviation (SD). Among women in the overweight/obesity group, a borderline mean BFG level and elevated triglycerides were observed, mirroring the pattern seen in men when examining the SD. These results are similar to those obtained in studies of Italian [26], Chinese [27], and Brazilian [28] adult populations, which tripled and even sextupled our study sample, with the exception of the results obtained with the 5CM. Only 17.2% of the total sample suffered from NCDs, such as hypertension, diabetes, insulin resistance, and hypercholesterolemia. Regarding healthy habits, higher weekly calorie intake and PA levels in MET min/week were diagnosed in the normal weight groups of men and women.

When analyzing the adipose mass measured using 5CM, it was evident that the group of men overweight or obese had no relationship with any of the studied parameters compared with the normal weight group, which had a relationship with total body BMD. However, when the 3CM model was used, the group of men with overweight or obesity was related to PA and adipose mass parameters, while the group of women with overweight or obesity was related to both 5CM and 3CM (Table 4 and Table 5). Comparing our results to current research on the adult population, the associations between fat mass and the studied parameters are diverse. Some authors report a direct and moderate correlation of fat mass with glucose, HDL cholesterol, and triglycerides, regardless of gender [26], while others found no association between fat mass and total body BMD when comparing samples from different ethnicities (African and Asian Indian) [29] and age groups [30]. In the latter, it was revealed that young women had an inverse and moderate correlation between fat mass and total body BMD; however, postmenopausal women showed a direct correlation. One explanation for this phenomenon is that adult women maintain more stable bone mass; however, over the years, as a result of the different hormonal changes, the fat mass has a protective effect on total body BMD as the adipocytes undergo the subcutaneous tissue transformation of androgens to estrogens, increasing the levels of circulating estrogens [30,36]. This affects the osteogenic differentiation of mesenchymal stem cells and the maturation of osteoblasts for bone formation, which, in turn, inhibits osteoclast formation and induces apoptosis for bone resorption [37]. In obese men, testosterone levels tend to fall, which is a risk factor for falls, especially in the elderly [37].

Muscle mass, or lean mass, is one of the most important components for maintaining a healthy life in adulthood and later in old age, as 10% of muscle mass is lost between the ages of 24 and 50, 30% between the ages of 50 and 80, and 1% per year over the age of 80 [38]. Aerobic exercises like walking, running, and cycling, as well as anaerobic strength exercises with loading and resistance during adolescence and adulthood, help build bone mass, slow bone loss due to aging and stop changes in the microarchitecture of bone tissue that happen with age [39]. This lowers the risk of fractures, especially in the hip for men and the humerus for women [40]. Our results show a direct and moderate relationship between muscle mass measured using 3CM and 5CM models and total body BMD in the overweight or obese groups (Table 4). We can infer that significantly higher muscle mass, similar to the normal weight group in both genders, leads to higher total body BMD [29,30].

In addition, in the group of men and women with overweight or obesity, muscle or lean mass presented a relationship with BFG (Table 4), similar to the results obtained in the study by Aparisi et al. (2018) (men: r = 0.333, *p* < 0.0001, and women: r = 0.495, *p* < 0.0001) [24]. The explanation for this association could be determined by the chronic inflammatory effect caused by excess malnutrition of adipokines in adipose tissue, causing insulin sensitivity in several tissues, specifically in muscle mass and liver [41]. Since muscle mass comprises approximately 40% of the body weight of an adult person and is responsible for the elimination and absorption of over 80% of glucose uptake throughout the body by insulin stimulation under normal conditions; therefore, when the muscle becomes resistant to the insulin released by the pancreas, it increases in BFG levels, causing pathologies such as insulin resistance or type 2 diabetes [42,43]. Although the percentage of participants who presented with these diseases is low, it is probable that they affect the correlation of the data.

Increased body weight, either through increased muscle and/or fat mass, can affect bone health. On the one hand, it can stimulate bone formation and decrease bone re-absorption, having a direct effect on the increase in BMD and BMC and the delay of diseases such as osteopenia and later osteoporosis in adulthood and later old age [27,40,43]. It is important to maintain a body weight within normal ranges since excess weight can generate an elevation of BMC, causing rigidity, fragility, and decreased bone flexibility, increasing the possibility of fractures [27,40,43]. Consistent with our findings, BMC measured using DXA is directly and strongly related to BMD in both sexes, both in the overweight/obesity and normal weight groups and in the normal weight group of 5CM men.

The skin and residual mass predict in kilograms the body surface area, in addition to the thickness and density of the skin, as well as the vital organs and viscera, respectively [23]. These were higher in the male group compared to the female group, independent of nutritional status, something that had already been found in the study by Kerr [23], which was confirmed in our study.

The 3CM and 5CM related the FFM to BFG and BMD in the group of overweight or obese men and women (Table 4). However, the 5CM did not show a relationship between FFM and BMD in the women’s group (Table 5). This agrees with the results obtained with muscle mass in our study and the physiological analysis with BMD and BFG [41,42,43] but is controversial to the results reported in a review of the literature [44], where no relationship or negative correlations were found between FFM and BFG in obese people.

### 4.1. Implications

The following implications can be drawn from this research. First, this research related 5CM to physiological variables, just like the indicators commonly used to assess body composition and nutritional status. Therefore, we identify the opportunity to use 5CM as an indicator of body composition in clinical and epidemiological practices rather than relying solely on BMI, WHR, and certain folds to identify obesity. We present the opportunity to generate cut-off points for the 5CM masses from the 3CM using the obtained data as the “gold standard”. This allowed us to analyze multivariate models adjusted (logistic regression) to the control and confounding variables, with the goal of evaluating the complex relationships of the explanatory variables studied. The goal was to get more population studies (with different research designs) to use 5CM so that the results could be compared in a more meaningful way. These studies should include metabolic and epigenetic factors that play a role in the unhealthy processes of obesity. The aim is to promote in this context. If future research supports it, public health should promote the use of 5CM to evaluate body composition and massify the corresponding certification.

### 4.2. Strengths and Limitations

Our research is the first in our country to study the 5CM as the most complete anthropometric method for the analysis of body composition in a clinical setting since it has only been used in general sports and elite fields, both nationally and internationally. At the same time, being able to compare this method to biochemical, nutritional, and bone variables and see their relationships and how these same variables behave similarly with body composition measured by DXA provides a relevant result between an indirect and a doubly indirect method of estimation. To reduce instrument and observer bias, the following considerations were considered: 1. use of DXA equipment and health personnel from a health center specializing in imaging; 2. use and calibration of the same equipment for body measurements and a certified anthropometrist; 3. nursing technician for blood sampling; 4. medical technologist for handling equipment in the biochemical analysis; 5. nutritionist for performing R24h. The databases to reduce the bias of the data processing were validated with the files of the participants before the statistical analysis.

Our study has some important limitations. First, the information bias of the participants in the administration of the R24h and the PA survey could affect the comparison and correlation of the variables studied. Second, the results cannot be extrapolated to the entire adult population of the La Araucanía region since they are only limited to adults aged 45 to 59 years old, excluding adults aged 25 to 44.9 years old because the sample was obtained from a cohort with a minimum age of 45 years, which was not an arbitrary decision, and only covers one commune out of a total of 32 in the entire region. Third, it is not possible to speak of causality in the study due to the type of design and the fact that the participants went through the measurement procedures only once. Fourth, the procedures, conducted in different locations, resulted in sample loss, leading to a disproportion between the groups. Fifthly, the classification of the case and control groups based on BMI may have influenced the obtained results, as both methods show a high fat mass in the male group, confounding the results.

## 5. Conclusions

In conclusion, as researchers, we highlight the use of the pentacompartmental model as a method to obtain the body composition of Chilean adults. In the clinical setting, its use would favor the more accurate obtaining of adipose mass (in kg and percentage) to evaluate the degree of obesity; muscle and bone mass as structural and strength indicators for a better quality of life; residual mass as a prediction of the weight of vital organs and viscera; and skin mass as an indicator of the body surface, as well as the thickness and density of the skin. In this study, it was possible to demonstrate the similarity of the results with the indirect method of 3CM obtained using DXA.

As a projection of this research, we emphasize the need for additional analysis on a larger population, as well as of the verification of the causal impact of the 5CM in a longitudinal study with different biological parameters measured across different age groups and ethnicities in order to obtain the representativeness of the whole region and improve the generalization of the findings.

## Figures and Tables

**Table 1:** Biosociodemographic, nutritional, and biochemical parameters in adult men and women with overweight/obesity and normal weight.

Parameters	Men (*n* = 52)					Women (*n* = 64)				
	Overweight/Obesity (*n* = 33)		Normal Weight (*n* = 19)		*p*-Value	Overweight/Obesity (*n* = 33)		Normal Weight (*n* = 31)		*p*-Value
	%	*n*	%	*n*		%	*n*	%	*n*	
**Biosociodemographic**										
Age (years old) ^1^	52.7	3.6	51.5	4.5	0.163	54.2	3.5	52.2	4.3	**0.023**
Location ^2^										
Rural	3.03	1	0.0	0	0.444	6.06	2	3.23	1	0.592
Urban	97.0	32	100.0	19		93.9	31	96.7	30	
Employment status ^2^										
Employed	57.6	19	52.6	10	0.328	36.4	12	48.4	15	0.658
Self-employed	33.3	11	42.1	8		18.2	6	19.6	6	
Unemployed	9.09	3	0.0	0		3.03	1	0.0	0	
Retired	0.0	0	5.3	1		0.0	0	0.0	0	
Housewife	0.0	0	0.0	0		42.4	14	32.26	10	
Marital status ^2^										
Married	69.7	23	47.4	9	0.116	66.8	22	41.9	13	0.334
Single	20.0	7	21.2	4		18.2	6	32.3	10	
Divorced	3.03	1	21.1	4		9.1	3	9.68	3	
Separated	3.03	1	10.5	2		3.03	1	9.68	3	
Cohabiting	3.03	1	0.0	0		3.12	1	6.5	2	
Widow(er)	0.0	0	0.0	0		0.0	0	0.0	0	
Number of NCDs ^3^	1	5	2	1	**0.025**	1	11	1	3	0.328
NCDs										
Hypertension	60.0	3	50.0	1	-	33.3	5	66.6	2	-
Diabetes	0.0	0	50.0	1	-	20.0	3	0.0	0	-
Hypercholesterolemia	0.0	0	0.0	0	-	20.0	3	33.3	1	-
Insulin resistance	40.0	2	0.0	0	-	26.6	4	0.0	0	-
Medications ^3^	1	5	4	1	**0.025**	2	11	1	3	0.115
**Nutritional parameters**										
Average R24h (kcal/week) ^1^	1762.1	501.2	1922.1	581.3	0.150	1388.3	239.9	1448.5	309.3	0.193
PA (MET min/week) ^3^	900	200–2280	1440	240– 2680	0.336	2160	1200–2720	1680	420– 2880	0.554
PAL ^4^										
Sedentary	42.4	14	31.6	6	0.738	15.2	5	29.0	9	0.311
Moderate	21.2	7	26.3	5		27.3	9	16.1	5	
High	36.4	12	42.1	8		57.6	19	54.8	17	
**Biochemical parameters ^1^**										
Blood fasting glucose (mg/dL)	104.8	27.5	89.6	9.68	**0.012**	96.9	34.4	83.9	7.02	**0.022**
Total Cholesterol (mg/dL)	229.9	51.6	207.1	44.6	0.057	205.6	46.5	224.8	52.9	0.064
LDL-Cholesterol (mg/dL)	147.8	47.5	122.6	34.7	**0.024**	133.2	36.0	127.1	42.9	0.271
HDL-Cholesterol (mg/dL)	61.4	18.9	51.9	20.5	**0.047**	67.8	14.3	73.5	19.4	0.091
Triglycerides (mg/dL)	197.9	111.5	101.2	48.1	**0.000**	142.5	89.9	90.7	59.6	**0.005**

Note. Age, average R24h, and biochemical parameters variables are expressed as mean and standard deviation; the number of NCDs and medication consumption are expressed as median and frequency; and PA is expressed as median and interquartile range. NCDs: noncommunicable chronic diseases; PA: physical activity; PAL: physical activity level; LDL: low-density lipoprotein; HDL: high-density lipoprotein. ^1^ *t*-Student test; ^2^ Fisher’s exact test; ^3^ Mann–Whitney U test; ^4^ Chi^2^ test.

**Table 2:** Anthropometric profile of normal-weight and overweight/obesity (Ov/Ob) adults by gender.

Anthropometric Profile	Men					Women				
	Ov/Ob		Normal Weight		*p*-Value	Ov/Ob		Normal Weight		*p*-Value
	Mean	SD	Mean	SD		Mean	SD	Mean	SD	
**Basic measurements**										
Weight (kg)	85.6	11.0	70.9	7.0	**<0.0000**	73.0	7.9	57.2	5.7	**<0.0000**
Height (cm)	171.0	6.7	172.5	6.3	0.213	156.0	5.7	157.7	5.8	0.123
Sitting Height (cm)	91.4	3.5	92.0	4.1	0.32	84.6	3.0	84.7	2.7	0.407
**Diameters (cm)**										
Biacromial	41.4	1.6	39.7	1.6	**0.0004**	37.2	1.6	36.0	1.5	**0.002**
Transverse Chest	31.0	2.0	29.5	1.4	**0.002**	27.7	1.5	25.8	1.5	**<0.0000**
Anteroposterior Chest	22.3	1.6	20.5	1.5	**0.0001**	19.2	1.7	17.4	1.3	**<0.0000**
Bi-iliocrestal	30.3	2.2	27.7	1.5	**<0.0000**	29.4	1.9	26.9	2.5	**<0.0000**
Humeral	7.2	0.3	7.02	0.3	**0.022**	6.4	0.4	6.1	0.3	**0.0001**
Femoral	9.9	0.6	9.69	0.5	**0.036**	9.2	0.5	8.7	0.4	**0.0003**
**Circumference (cm)**										
Head	57.2	1.3	56.1	1.3	**0.002**	54.5	1.9	53.9	1.2	0.077
Relaxed arm	33.4	2.4	29.6	1.4	**<0.0000**	31.3	1.9	27.5	1.9	**<0.0000**
Maximum forearm	28.6	1.8	26.2	1.2	**<0.0000**	25.0	1.2	23.1	1.2	**<0.0000**
Mesoesternal thoracic	107.0	5.8	98.9	4.3	**<0.0000**	98.7	5.7	88.9	5.3	**<0.0000**
Waist (minimum)	99.2	7.4	86.0	4.7	**<0.0000**	94.9	9.9	77.8	6.2	**<0.0000**
Hip (maximum)	101.3	6.0	93.4	4.2	**<0.0000**	104.9	7.8	92.8	4.4	**<0.0000**
Thigh (medial)	54.1	4.1	49.4	3.2	**<0.0000**	51.5	3.5	46.8	3.1	**<0.0000**
Calf (maximum)	38.5	2.8	35.7	2.3	**0.0003**	36.6	2.2	33.1	1.9	**<0.0000**
**Skinfolds (mm)**										
Triceps	13.6	3.9	10.2	2.6	**0.0007**	26.0	5.4	18.0	3.9	**<0.0000**
Subscapular	23.0	6.6	14.1	5.4	**<0.0000**	30.0	7.6	16.8	5.0	**<0.0000**
Supraspinous	16.8	6.0	11.8	2.7	**0.0005**	28.4	9.1	15.2	6.5	**<0.0000**
Abdominal	28.0	5.8	22.6	4.4	**0.0005**	37.0	10.9	21.5	5.9	**<0.0000**
Medial Thigh	11.8	3.7	11.2	2.8	0.244	27.0	8.4	19.3	4.8	**<0.0000**
Leg (maximum)	7.2	2.6	6.2	2.3	0.081	18.1	6.2	10.6	3.5	**<0.0000**

All analyses were performed with the *t*-Student test mean comparison method. SD: standard deviation.

**Table 3:** A comparison of WHR, body composition, and bone mineral density among normal weight and overweight/obesity (Ov/Ob) adults by gender.

Variables	Men					Women				
	Ov/Ob		Normal Weight		*p*-Value	Ov/Ob		Normal Weight		*p*-Value
	Mean	SD	Mean	SD		Mean	SD	Mean	SD	
WHR ^1^	0.98	0.04	0.92	0.05	**<0.0000**	0.90	0.06	0.84	0.05	**<0.0000**
WHR categories ^2^										
Low + Healthy	42.9	12	84.2	16	**0.001**	9.09	3	29.0	9	**0.041**
Excess fat + obese	63.6	21	15.8	3		90.9	30	71.0	22	
**Body composition**										
5CM ^1^										
Adipose Mass (kg)	22.0	4.14	18.5	3.18	**0.0011**	26.6	5.38	18.5	3.45	**<0.0000**
Adipose Mass (%)	25.6	3.47	25.9	3.04	0.407	36.3	4.55	32.2	4.16	**0.0002**
Muscle Mass (kg)	39.0	5.78	32.2	3.63	**0.0001**	26.5	2.96	22.7	3.14	**<0.0000**
Muscle Mass (%)	45.4	3.31	45.3	2.85	0.466	36.6	3.79	39.6	4.04	**0.002**
Bone Mass (kg)	9.28	1.22	7.84	0.93	**<0.0000**	7.26	0.78	6.11	0.67	**<0.0000**
Bone Mass (%)	10.9	0.70	11.0	0.81	0.759	9.97	0.77	10.8	1.14	**0.0009**
Skin Mass (kg)	3.88	0.34	3.60	0.28	**0.002**	3.42	0.22	3.13	0.22	**<0.0000**
Skin Mass (%)	4.54	0.24	5.09	0.23	**<0.0000**	4.71	0.33	5.49	0.36	**<0.0000**
Residual Mass (kg)	11.7	1.83	9.03	0.99	**<0.0000**	9.11	1.45	6.84	1.09	**<0.0000**
Residual Mass (%)	13.6	0.80	12.7	1.03	**0.0007**	12.5	1.59	11.9	1.56	0.086
Fat-free Mass (kg)	63.8	8.68	52.6	5.10	**<0.0000**	46.3	4.44	38.8	4.01	**<0.0000**
Fat-free Mass (%)	74.4	3.47	74.1	3.05	0.401	63.8	4.54	67.8	4.17	**0.0002**
3CM ^1^										
Fat Mass (kg)	25.8	6.51	16.8	3.99	**<0.0000**	31.4	6.45	20.0	4.41	**<0.0000**
Fat Mass (%)	29.5	5.24	22.9	4.56	**<0.0000**	43.0	5.14	34.1	5.48	**<0.0000**
Lean Mass (kg)	57.5	7.07	52.4	5.37	**0.004**	38.8	3.61	36.0	3.66	**0.001**
Lean Mass (%)	66.7	4.93	72.2	5.1	**0.0001**	53.8	4.92	62.2	5.30	**<0.0000**
BMC Complete Body (Kg)	3.21	0.44	3.00	0.52	0.07	2.37	0.30	2.16	0.33	**0.005**
BMC Complete Body (%)	3.73	0.45	4.11	0.40	**0.0012**	3.26	0.41	3.71	0.43	**<0.0000**
Fat-free Mass (kg)	60.7	7.42	55.9	5.65	**0.009**	41.1	3.78	38.1	3.88	**0.0013**
Fat-free Mass (%)	70.4	5.23	77.1	4.56	**<0.0000**	57.1	5.14	65.9	5.49	**<0.0000**
**BMD (g/cm^2^)** ^1^	1.29	0.09	1.23	0.12	**0.033**	1.16	0.09	1.11	0.10	**0.02**
BMD Categories ^2^										
Normal	97.0	32	84.2	16	0.132	93.9	31	87.1	27	0.504
Osteopenia	3.0	1	10.5	2		6.06	2	9.68	3	
Osteoporosis	0.0	0	5.26	1		0.0	0	3.23	1	

Note: The variables WHR and BMD categories are expressed in percentage and frequency. SD: standard deviation; WHR: waist-to-hip ratio; 5CM: pentacompartmental model; 3CM: tricompartmental model; BMC: bone mineral content; BMD: bone mineral density. ^1^ *t*-Student test; ^2^ Fisher’s exact test.

**Table 4:** Relationship between body composition by 5CM, biochemical and nutritional parameters, and BMD in normal weight (NW) and overweight/obesity (Ov/Ob) adults by gender.

	Body Composition 5CM in Men											
	Adipose Mass		Muscle Mass		Bone Mass		Skin Mass		Residual Mass		FFM	
	Ov/Ob	NW	Ov/Ob	NW	Ov/Ob	NW	Ov/Ob	NW	Ov/Ob	NW	Ov/Ob	NW
BFG	0.051	0.353	**0.477** **	−0.068	**0.384** *	0.115	**0.433** *	0.146	**0.502** **	**0.465** *	**0.494** **	0.071
TC	0.190	0.112	0.166	0.374	0.102	0.229	0.160	0.208	0.181	−0.136	0.168	0.293
LDL-C	0.153	0.063	0.085	0.152	0.022	0.086	0.105	0.060	0.164	−0.215	0.098	0.086
HDL-C	0.268	−0.343	0.148	0.103	0.121	0.195	0.146	0.018	0.201	−0.141	0.164	0.083
TRIG	0.040	−0.087	0.260	−0.096	0.125	−0.136	0.125	−0.126	0.116	0.186	0.244	0.064
BMD	−0.241	**0.472** *	**0.461** **	**0.691** ***	0.201	**0.700** ***	0.227	**0.635** **	0.135	**0.487** *	**0.372** *	**0.749** ***
R24h	−0.138	−0.052	0.209	−0.085	0.173	−0.003	0.147	−0.066	0.059	0.038	0.181	−0.058
PA	−0.329	−0.008	−0.078	0.206	−0.195	−0.068	−0.147	0.136	−0.200	−0.220	−0.099	−0.004
	**Body Composition 5CM in Women**											
	**Adipose Mass**		**Muscle Mass**		**Bone Mass**		**Skin Mass**		**Residual Mass**		**FFM**	
	**Ov/Ob**	**NW**	**Ov/Ob**	**NW**	**Ov/Ob**	**NW**	**Ov/Ob**	**NW**	**Ov/Ob**	**NW**	**Ov/Ob**	**NW**
BFG	0.196	0.034	**0.432** *	0.258	0.359	0.056	0.162	0.291	0.268	−0.211	**0.453** **	0.170
TC	−0.097	0.229	−0.223	0.201	−0.088	−0.031	−0.160	**0.376** *	0.046	0.253	−0.157	0.242
LDL-C	0.044	0.171	−0.191	0.063	−0.019	0.008	0.065	0.326	0.229	0.100	−0.047	0.096
HDL-C	−0.087	−0.092	−0.241	0.024	0.009	**−0.370** *	−0.105	−0.041	−0.134	−0.011	0.347	−0.049
TRIG	0.235	**0.371** *	0.114	0.294	**0.344** *	0.289	−0.001	**0.489** **	0.345	0.150	0.250	0.347
BMD	0.147	0.034	**0.349** *	**0.441** *	0.137	−0.097	0.174	0.318	0.047	0.291	0.281	**0.427** *
R24h	0.338	0.133	−0.257	−0.198	0.022	−0.055	−0.127	−0.157	−0.318	0.053	−0.278	−0.158
PA	**−0.459** *	−0.067	−0.038	0.146	−0.292	−0.013	−0.256	−0.048	**−0.526** **	0.023	−0.238	0.078

Note. Pearson’s r test, with the exception of physical activity, and Spearman’s rank correlation test were used. * *p* < 0.05; ** *p* < 0.01; *** *p* < 0.001. Ov/Ob: overweight/obesity; NW: normal weight; 5CM: pentacompartmental model; FFM: fat-free mass; BFG: blood fasting glucose; TC: total cholesterol; LDL-C: low-density lipoprotein cholesterol; HDL-C: high-density lipoprotein cholesterol; TRIG: triglycerides; BMD: bone mineral density; R24h: average 24-h reminder; PA: physical activity.

**Table 5:** Relationship among body composition by 3CM, biochemical and nutritional parameters, and BMD in normal weight (NW) and overweight/obesity (Ov/Ob) adults by gender.

	Body Composition 3CM in Men							
	Fat Mass		Lean Mass		BMC		FFM	
	Ov/Ob	NW	Ov/Ob	NW	Ov/Ob	NW	Ov/Ob	NW
BFG	0.185	0.316	**0.394** *	−0.093	0.228	0.079	**0.390** *	−0.008
TC	0.183	0.120	0.202	0.310	0.098	0.106	0.198	0.261
LDL-C	0.201	0.101	0.083	0.062	−0.093	−0.102	0.074	0.051
HDL-C	0.256	−0.350	0.102	0.240	−0.066	−0.091	0.094	0.129
TRIG	0.084	0.016	0.276	−0.164	0.323	−0.252	0.284	−0.177
BMD	−0.207	0.333	**0.411** *	**0.634** ***	**0.832** ****	**0.932** ****	**0.442** **	**0.771** ****
R24h	−0.114	−0.119	0.220	−0.046	0.245	−0.071	0.244	−0.020
PA	**−0.493** **	0.058	0.004	0.308	0.218	0.050	0.007	0.102
	**Body Composition 3CM in Women**							
	**Fat Mass**		**Lean Mass**		**BMC**		**FFM**	
	**Ov/Ob**	**NW**	**Ov/Ob**	**NW**	**Ov/Ob**	**NW**	**Ov/Ob**	**NW**
BFG	0.248	−0.073	**0.504** **	0.109	0.231	0.270	**0.499** **	0.125
TC	−0.122	0.161	−0.106	0.314	−0.018	**0.372** *	−0.188	0.327
LDL-C	0.046	0.146	−0.194	0.117	−0.021	0.238	−0.187	0.130
HDL-C	−0.144	−0.108	−0.196	0.056	0.068	−0.074	−0.182	0.047
TRIG	0.337	**0.390** *	0.037	0.243	0.152	0.217	0.047	0.247
BMD	0.096	−0.086	**0.408** *	**0.533** **	**0.894** ****	**0.830** ****	**0.461** **	**0.572** ***
R24h	0.061	0.120	0.082	−0.287	0.006	−0.101	0.078	−0.279
PA	**−0.421** *	−0.079	−0.163	0.317	0.107	0.139	−0.155	0.325

Note. Pearson’s r test, with the exception of physical activity, and Spearman’s rank correlation test were used. * *p* < 0.05; ** *p* < 0.01; *** *p* < 0.001; **** *p* < 0.0000. Ov/Ob: overweight/obesity; NW: normal weight; 3CM: tricompartmental model; FFM: fat-free Mass; BFG: blood fasting glucose; TC: total cholesterol; LDL-C: low-density lipoprotein cholesterol; HDL-C: high-density lipoprotein cholesterol; TRIG: triglycerides; BMD: bone mineral density; R24h: average 24-h reminder; PA: physical activity.

## Data Availability

Data are available by request due to licensing reasons.

## Supplementary Materials

The following supporting information can be downloaded at https://www.mdpi.com/article/10.3390/nu16111559/s1, Figure S1 (a–g). Correlation of body composition by 5CM and biochemical and nutritional parameters and BMD in adult men with overweight/obesity. Figure S2 (a–f). Correlation of body composition by 3CM and biochemical and nutritional parameters and BMD, in adult men with overweight/obesity. Figure S3 (a–f). Correlation of body composition by 5CM and biochemical and nutritional parameters and BMD in adult women with overweight/obesity. Figure S4 (a–f). Correlation of body composition by 3CM and biochemical and nutritional parameters and BMD, in adult women with overweight/obesity.

## References

[1] Swinburn B.A., Sacks G., Hall K.D., McPherson K., Finegood D.T., Moodie M.L., Gortmaker S.L. (2011). The Global Obesity Pandemic: Shaped by Global Drivers and Local Environments. Lancet.

[2] World Health Organization (WHO) (2024). Obesity and Overweight. https://www.who.int/news-room/fact-sheets/detail/obesity-and-overweight.

[3] Hruby A., Manson J.A.E., Qi L., Malik V.S., Rimm E.B., Sun Q., Willett W.C., Hu F.B. (2016). Determinants and Consequences of Obesity. Am. J. Public Health.

[4] Marti A., Moreno-Aliaga M.J., Hebebrand J., Martínez J.A. (2004). Genes, Lifestyles and Obesity. Int. J. Obes..

[5] González-Muniesa P., Mártinez-González M.A., Hu F.B., Després J.P., Matsuzawa Y., Loos R.J.F., Moreno L.A., Bray G.A., Martinez J.A. (2017). Obesity. Nat. Rev. Dis. Primers.

[6] Petermann-Rocha F., Martínez-Sanguinetti M.A., Villagrán M., Ulloa N., Nazar G., Troncoso-Pantoja C., Garrido-Méndez A., Mardones L., Lanuza F., Leiva A.M. (2020). From a Global View to the Chilean Context: Which Factors Have Influenced the Development of Obesity in Chile? (Chapter 1). Rev. Chil. Nutr..

[7] Organization for Economic Co-operation and Development (OECD) (2019). OECD Studies on Public Health: Chile, towards a Healthier Future. Evaluation and Recommendationst.

[8] Ministerio de Salud (MINSAL) (2019). Encuesta Nacional de La Salud 2016–2017: Informe Final.

[9] Andreoli A., Garaci F., Cafarelli F.P., Guglielmi G. (2016). Body Composition in Clinical Practice. Eur. J. Radiol..

[10] Domaradzki J., Koźlenia D. (2022). The Performance of Body Mass Component Indices in Detecting Risk of Musculoskeletal Injuries in Physically Active Young Men and Women. PeerJ.

[11] Kuriyan R. (2018). Body Composition Techniques. Indian J. Med. Res..

[12] Cummings S.R., Bates D., Black D.M. (2002). Clinical Use of Bone Densitometry: Scientific Review. JAMA.

[13] Lorente Ramos R.M., Azpeitia Armán J., Arévalo Galeano N., Muñoz Hernández A., García Gómez J.M., Gredilla Molinero J. (2012). Dual Energy X-ray Absorptimetry: Fundamentals, Methodology, and Clinical Applications. Radiologia.

[14] Shiel F., Persson C., Furness J., Simas V., Pope R., Climstein M., Hing W., Schram B. (2018). Dual Energy X-ray Absorptiometry Positioning Protocols in Assessing Body Composition: A Systematic Review of the Literature. J. Sci. Med. Sport..

[15] González Jiménez E. (2013). Body Composition: Assessment and Clinical Value. Endocrinol. Nutr. (Engl. Ed.).

[16] Rosales Ricardo Y. (2012). Anthropometry in the diagnosis of obese patients; A review. Nutr. Hosp..

[17] Wells J.C.K. (2014). Commentary: The Paradox of Body Mass Index in Obesity Assessment: Not a Good Index of Adiposity, but Not a Bad Index of Cardio-Metabolic Risk. Int. J. Epidemiol..

[18] Frisancho A.R. (1990). Anthropometric Standards for the Assessment of Growth and Nutritional Status.

[19] Henneberg M., Ulijaszek S.J. (2010). Body Frame Dimensions Are Related to Obesity and Fatness: Lean Trunk Size, Skinfolds, and Body Mass Index. Am. J. Hum. Biol..

[20] Holway F., Boullosa M.B., Peniche Z.C. (2011). Chapter 8: Composición Corporal En Nutrición Deportiva. Nutrición Aplicada al Deporte.

[21] Ross W., Wilson N. (1974). A stratagem for proportional growth assessment. Acta Paediatr. Belg..

[22] Drinkwater D., Martin A., Ross W., Clarys J.P., Day J.A.P. (1984). Validation by cadaver dissection of Matiegka’s equations for the anthropometric estimation of anatomical body composition in adults humans. Perspectives in Kinanthropometry.

[23] Kerr D. (1988). An Anthropometric Method for Fractionation of Skin, Adipose, Bone, Muscle, and Residual Tissue Masses, in Males and, Females Age 6 to 77 Years. Master’s Thesis.

[24] Gajardo R., Gómez C., Flández J., Martínez S., Monrroy M. (2013). Anthropometric Profile of Chilean Junior Rowers. Int. J. Morphol..

[25] Yáñez-Sepúlveda R., Díaz-Barrientos S., Montiel-González S., Zavala-Crichton J.P. (2018). Anthropometric Characteristics, Body Composition and Somatotype in Elite Junior ITF Tennis Players from South America. Int. J. Morphol..

[26] Aparisi Gómez M.P., Ponti F., Mercatelli D., Gasperini C., Napoli A., Battista G., Cariani S., Marchesini G., Bazzocchi A. (2019). Correlation between DXA and Laboratory Parameters in Normal Weight, Overweight, and Obese Patients. Nutrition.

[27] Zhang Y., Jia X., Liu X., An W., Li J., Zhang W. (2022). Relationship between Different Body Composition and Bone Mineral Density in Qinhuangdao City. Rev. Assoc. Med. Bras..

[28] Segheto K.J., Juvanhol L.L., de Carvalho C.J., da Silva D.C.G., Kakehasi A.M., Longo G.Z. (2019). Factors Associated with Bone Mineral Content in Adults: A Population-Based Study. Einstein.

[29] George J.A., Micklesfield L.K., Norris S.A., Crowther N.J. (2014). The Association between Body Composition, 25(OH)D, and PTH and Bone Mineral Density in Black African and Asian Indian Population Groups. J. Clin. Endocrinol. Metab..

[30] Cheng Q., Zhu Y.X., Zhang M.X., Li L.H., Du P.Y., Zhu M.H. (2012). Age and Sex Effects on the Association between Body Composition and Bone Mineral Density in Healthy Chinese Men and Women. Menopause.

[31] Norton K. (2018). Chapter 4 Standards for Anthropometry Assessment. Kinanthropometry and Exercise Physiology.

[32] Gibson R.S. (2005). Principles of Nutritional Assessment.

[33] WHO (2021). Global Physical Activity Questionnaire (GPAQ). https://www.who.int/es/publications/m/item/global-physical-activity-questionnaire.

[34] Blanton C.A., Moshfegh A.J., Baer D.J., Kretsch M.J. (2006). The USDA Automated Multiple-Pass Method Accurately Estimates Group Total Energy and Nutrient Intake. J. Nutr..

[35] Cerda R., Barrera C., Arena M., Bascuñán K., Jiménez G. (2010). Atlas Fotográfico de Alimentos y Preparaciones Típicas Chilenas: Encuesta Nacional de Consumo Alimentario 2010.

[36] Emmanuelle N.E., Marie-Cécile V., Florence T., Jean-François A., Françoise L., Coralie F., Alexia V. (2021). Critical Role of Estrogens on Bone Homeostasis in Both Male and Female: From Physiology to Medical Implications. Int. J. Mol. Sci..

[37] Fischer V., Haffner-Luntzer M. (2022). Interaction between bone and immune cells: Implications for postmenopausal osteoporosis. Semin. Cell Dev. Biol..

[38] Deschenes M.R. (2004). Effects of aging on muscle fibre type and size. Sport. Med..

[39] Benedetti M.G., Furlini G., Zati A., Mauro G.L. (2018). The Effectiveness of Physical Exercise on Bone Density in Osteoporotic Patients. BioMed Res. Int..

[40] Kupisz-Urbańska M., Stuss M., Kuryłowicz A., Jankowski P., Pilz S., Sewerynek E., Marcinowska-Suchowierska E. (2022). Fracture Risk in Obesity: A Narrative Review. Endokrynol. Pol..

[41] Wu H., Ballantyne C.M. (2017). Skeletal Muscle Inflammation and Insulin Resistance in Obesity. J. Clin. Investig..

[42] Merz K.E., Thurmond D.C. (2021). Role of Skeletal Muscle in Insulin Resistance and Glucose Uptake. Compr. Physiol..

[43] López-Gómez J.J., Pérez Castrillón J.L., de Luis Román D.A. (2016). Influencia de La Obesidad Sobre El Metabolismo Óseo. Endocrinol. Nutr..

[44] Perreault K., Lagacé J.C., Brochu M., Dionne I.J. (2016). Association between Fat Free Mass and Glucose Homeostasis: Common Knowledge Revisited. Ageing Res. Rev..

