# Supplementary material for: Body Composition and Its Interaction with Bone Mineral Density and Biochemical and Nutritional Parameters in Chilean Adults with Overweight/Obesity and Normal Weight
Source: Nutrients. 2024 May 21;16(11):1559. doi: 10.3390/nu16111559 (PMC11173841; doi:10.3390/nu16111559)
Supplement: Supplementary file 1 [file nutrients-16-01559-s001.zip › nutrients-2905750-supplementary.pdf]

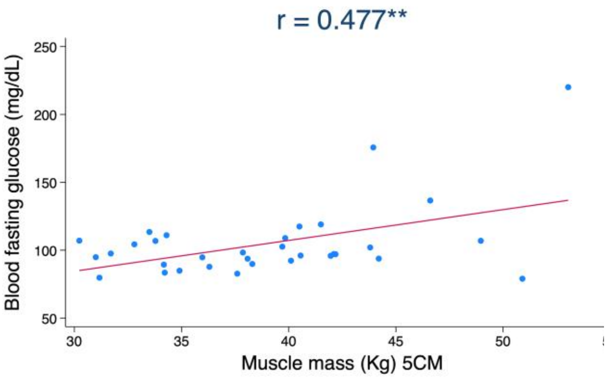

(a)

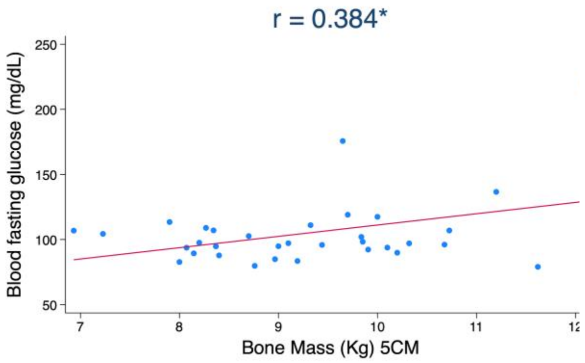

(b)

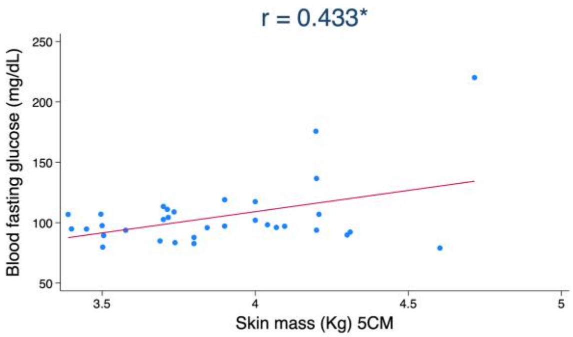

(c)

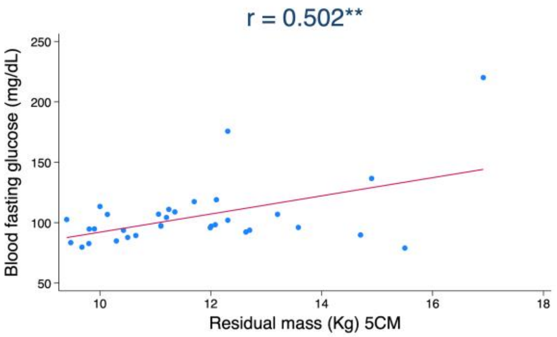

(d)

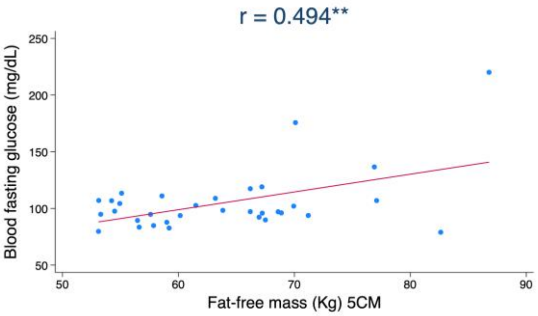

(e)

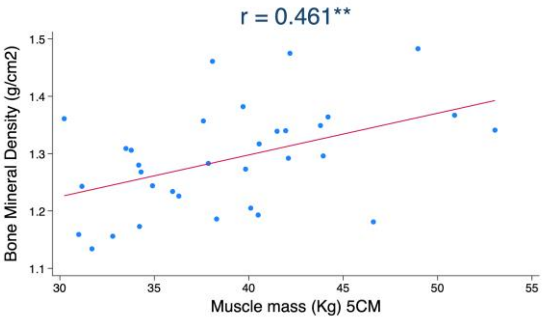

(f)

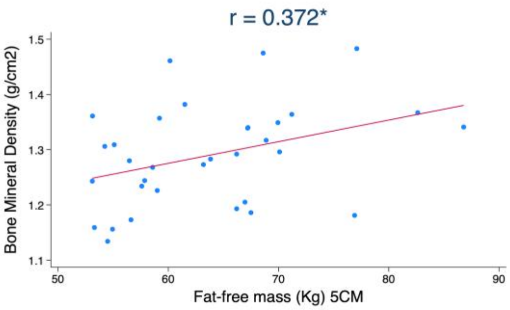

(g)

Figure S1 (a-g). Correlation of body composition by 5CM and biochemical and nutritional parameters and BMD, in adult men with overweight/obesity.

Note. Pearson's r test, exception physical activity Spearman's rank correlation test was used. *p < 0.05; **p < 0.01. Abbreviations. 5CM: Pentacompartmental Model.

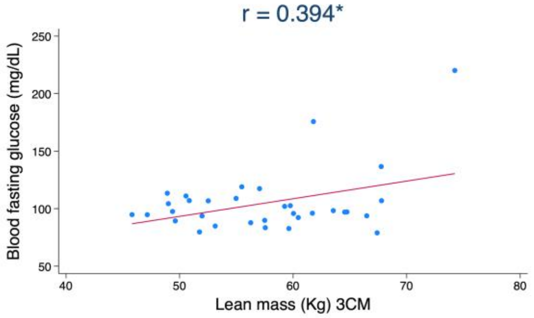

(a)

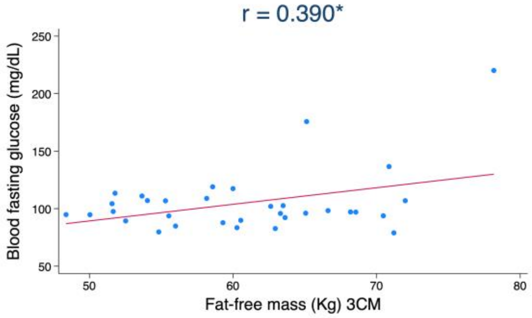

(b)

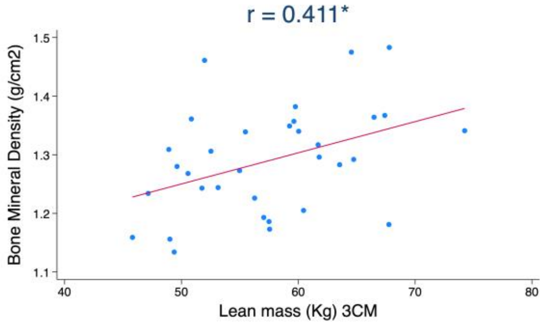

(c)

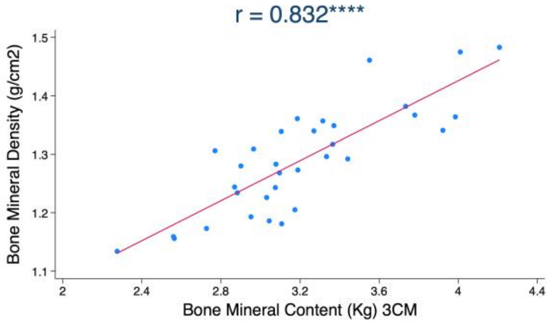

(d)

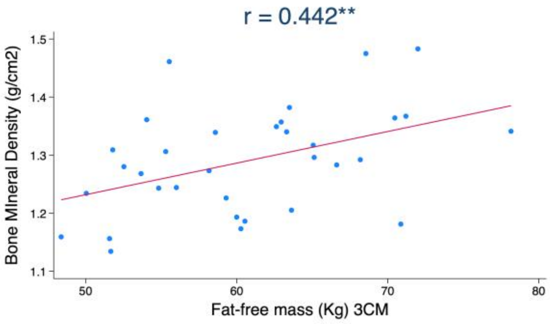

(e)

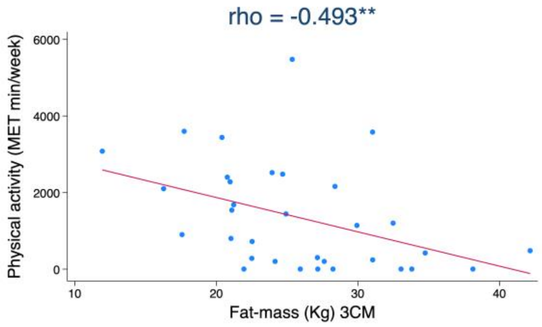

(f)

Figure S2 (a-e). Correlation of body composition by 3CM and biochemical and nutritional parameters and BMD, in adult men with overweight/obesity.

Note. Pearson's r test, exception physical activity Spearman's rank correlation test was used. *p<0.05; **p<0.01; ****p<0.0000.

Abbreviations. 3CM: Tricompartmental Model.

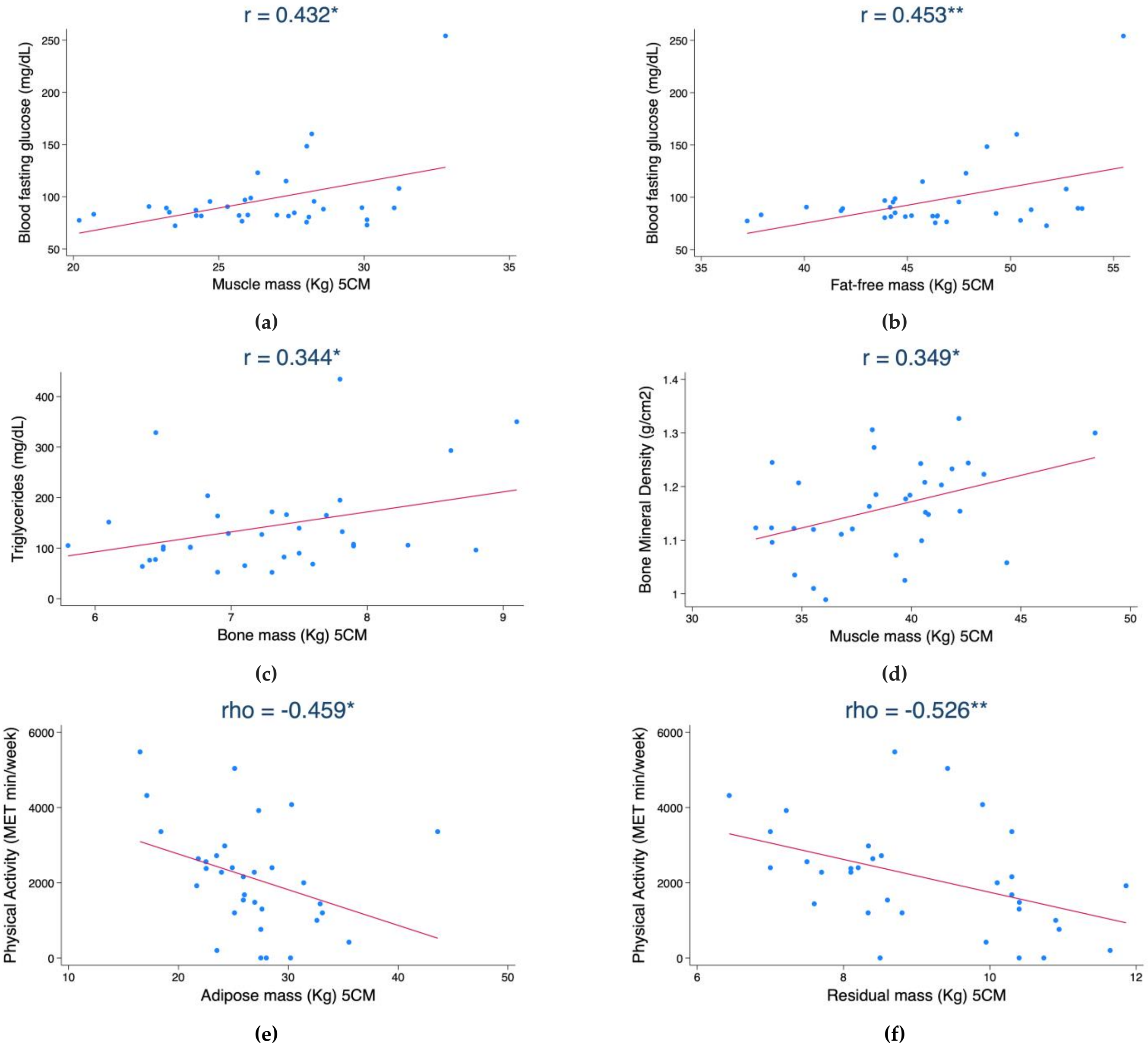

Figure S3 (a-f). Correlation of body composition by 5CM and biochemical and nutritional parameters and BMD, in adult women with overweight/obesity.

Note. Pearson's r test, exception physical activity Spearman's rank correlation test was used. *p<0.05; **p<0.01.
Abbreviations. 5CM: pentacompartmental model.

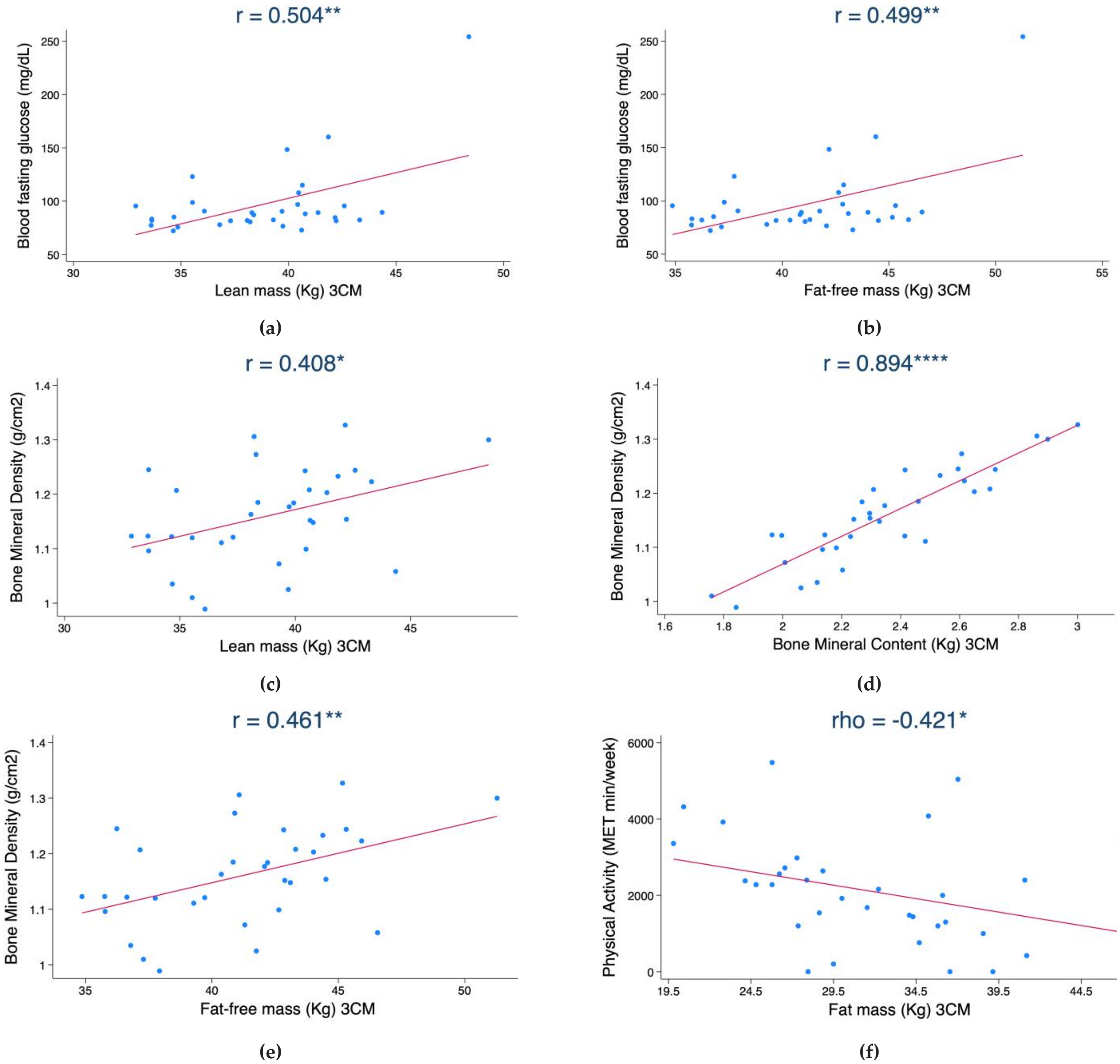

Figure S4 (a-f). Correlation of body composition by 3CM and biochemical and nutritional parameters and BMD, in adult women with overweight/obesity.

Note. Pearson's r test, exception physical activity Spearman's rank correlation test was used. *p<0.05; **p<0.01; ****p<0.0000.

Abbreviations. 3CM: tricompartmental model.
